# Salmonella enterica serovar Typhimurium inhibits the innate immune response and promotes apoptosis in a ribosomal/TRP53-dependent manner in swine neutrophils

**DOI:** 10.1186/s13567-020-00828-3

**Published:** 2020-08-27

**Authors:** Tinghua Huang, Caiyun Jiang, Min Yang, Hong Xiao, Xiali Huang, Lingbo Wu, Min Yao

**Affiliations:** College of Animal Science, Yangtze University, 434025 Jingzhou, Hubei China

**Keywords:** porcine, neutrophils, deep-sequencing, *Salmonella*, RPL39, RPL9, TRP53

## Abstract

Neutrophils are the first barriers for resisting the invasion, proliferation, and damage caused by *Salmonella* Typhimurium. However, the mechanisms that control this resistance are not completely understood. In this study, we established an in vitro *Salmonella* infection model in porcine neutrophils, and analyzed the cellular transcriptome by deep sequencing and flow cytometry. The results showed that ribosomal gene transcription was inhibited, and two of these genes, RPL39 and RPL9, were related to *TRP53* activation. Furthermore, several important innate immunity genes were also inhibited. Knock-down of RPL39 and RPL9 by siRNA caused an approximate fourfold up-regulation of TRP53. Knock-down of RPL39 and RPL9 also resulted in a significant down-regulation of IFNG and TNF, indicating an inhibition of the innate immune response. Silencing of RPL39 and RPL9 also resulted in the up-regulation of FAS, RB1, CASP6, and GADD45A, which play roles in cell cycle arrest and apoptosis. Neutrophils were either first treated with RPL39 siRNA, RPL9 siRNA, TRP53 activator, or TRP53 inhibitor, and then infected with *Salmonella*. Knock-down of RPL39 and RPL9, or treatment with TRP53 activator, can increase the intracellular proliferation of *Salmonella* in neutrophils. We speculate that much of the *Salmonella* virulence can be attributed to the enhancement of cell cycle arrest and the inhibition of the innate immune response, which allows the bacteria to successfully proliferate intracellularly.

## Introduction

*Salmonella enterica* serovar Typhimurium is a Gram-negative facultative intracellular bacterium that colonizes both animals and humans [1]. Pigs carrying *Salmonella* can shed bacteria on a farm, which may subsequently lead to the bacterial contamination of pig carcasses in a slaughterhouse, thereby posing a serious threat to the swine industry [2]. A *Salmonella* infection is a dynamic process that involves the interaction between various immune cells and the bacteria. Burton et al. demonstrated through single cell analysis and computer modeling that *Salmonella* bacteria can survive inside macrophages but are partially killed when engulfed by neutrophils and inflammatory monocytes [3]. The main mechanisms by which bacteria are effectively killed are through the production of a lethal concentration of reactive oxygen species (ROS) and hypochloric acid in phagocytes [4]. When *Salmonella* is engulfed by macrophages, the bacteria release a series of virulence factors through the type III secretion system, which modulates lysosome and vesicle maturation. This may reduce the pre-mature macrophage antibacterial response and may provide an ecological niche for *Salmonella* replication [5]. In the peripheral blood, neutrophils are the most abundant type of leukocytes. Recent studies have shown that the killing activity of neutrophils infected with *Salmonella* was found to decrease significantly after 2 h, but the overall bacterial proliferation was not significantly different from *Salmonella* in primary macrophages [6–8].

In the early stages of infection (24 to 48 h), *Salmonella* interacts with most phagocytic and non-phagocytic immune cells, including B-lymphocytes, T-lymphocytes, neutrophils, monocytes, and dendritic cells, however, *Salmonella* bacteria have never been detected in mature macrophages [5]. In the cells containing *Salmonella*, neutrophils represent the largest and most active population of cells that may contain *Salmonella* [5]. Similar to the behavior within macrophages, *Salmonella* mainly interacts with neutrophils through the type III secretion system and weakens the bactericidal activity, allowing *Salmonella* to survive and proliferate within neutrophils [5].

Ribosomes translate genetic information stored in mRNA into peptides in several steps, including initiation, extension, termination, and circulation. Previous studies have reported *Salmonella*-induced transcriptional inhibition of ribosomal genes in the peripheral blood of piglets infected with *Salmonella enterica* serovar Typhimurium [9]. This has been confirmed by high-throughput sequencing experiments [10]. Following infection with *Salmonella*, peripheral blood collected from piglets demonstrated a significant down-regulation of 55 ribosomal protein genes [10]. This involved 6 translation-related signaling pathways, which suggests that *Salmonella* infection modulates multiple key steps in the gene translation process. While it has been shown that peripheral blood collected from piglets infected with *Salmonella* contains many different types of hemocytes, the specific cells that are responsible for the gene expression responses remain unknown. In this study, the transcriptome of neutrophils infected with *Salmonella* was determined through deep sequencing, and we proposed a mechanism for *Salmonella* modulation of the host transcriptome and intracellular survival.

## Materials and methods

### Neutrophil isolation and infection

Peripheral blood samples were collected from 20 healthy crossbreed (Duroc × Landrace × Yorkshire) piglets obtained from a commercial swine farm in Jingzhou, China without sacrificing the animals (ten males and ten females, four weeks old). Piglets were randomly selected from ten litters (2 animals for each litter) and then co-housed together in climate-controlled facilities. Prior to blood collection, the feces were tested negative for *Salmonella* three times. *Salmonella* was quantified from feces by direct counting using bacteriological methods, as described by Uthe et al. [11]. The sample collection was approved by the Animal Care and Use Committee of Hubei Province (China, YZU-2018–0031). Neutrophils were isolated by density gradient centrifugation using Ficoll-Paque™ media (GE Healthcare Life Sciences, Shanghai, China), as per the manufacturer’s instructions. Luria broth and M9 minimal medium were used to culture *Salmonella* bacteria. The neutrophils were treated with 10^6^ colony forming units (cfu) of *Salmonella enterica* serovar Typhimurium (ATCC® 14,028™) as previously described (multiplicity of infection = 100:1) [10, 12]. Cells infected with *Salmonella* were collected at zero or eight hours post infection (hpi). Total RNA was prepared from the neutrophil solutions using the RNeasy Mini Kit as per the manufacturer’s instructions (Qiagen, Cat. no. 74104). The RNA quantity and quality were determined using the Agilent 2100 Bioanalyzer (Agilent Technologies, Santa Clara, CA, USA). Samples with an RNA Integrity Number (RIN) < 7 or a yield < 10 μg were excluded from the experiment.

### Deep sequencing, statistical analysis, and functional annotation

A total of six samples were collected from each group (three samples from male animals and three samples from female animals) at zero and eight hpi, and were randomly selected for transcriptome analysis. Sequencing libraries were prepared using the Illumina TruSeq RNA Sample Preparation kit according to the manufacturer’s protocol (Illumina Inc., USA). Sequencing was conducted using an Illumina HiSeq 2000 (Illumina Inc., USA) by single-read sequencing with a read length of 50 bp. The obtained raw reads were filtered by removing the low-quality reads, i.e., reads containing unknown bases (N) and reads containing bases with a quality value ≤ 5. Bowtie1 [13] was used to map clean reads to the reference gene set, which was extracted from the NCBI reference sequence database [14]. The LIMMA R package [15] was used to calculate read counts per gene per length, which was used to compare the differences in gene expression between samples collected at zero and eight hpi. The criteria for differentially expressed genes were controlled as FDR (false discovery rate) ≤ 0.05 and fold change ≥ 1.5 or ≤ 0.67 (standard).

The most current porcine gene annotation (*Sus scrofa* assembly 11.1, gene 99) was used to assign the transcripts to mouse RefSeq according to the dual best match method. InnateDB [16] was used to identify significantly up-regulated pathways between zero and eight hpi and DAVID [17] was used to annotate the genes. The GEREA bioinformatics tool [18] was used to identify the overrepresented regulator genes. All data discussed in this study have been deposited into the NCBI GEO database [19] under accession number GSE148236.

### Measuring *TRP53* levels following neutrophil infection with *Salmonella* using flow cytometry and Real-time PCR

Fifteen out of the twenty neutrophil samples (with the best yield of cell counts) were randomly selected and divided into five groups (after 2 h of recovering). Each group was used in separate experiments. Two groups were treated with RPL39 or RPL9 siRNA (10 nM, Nicolas’s sequence [20]) using Monceaux’s protocol [21]. Another two groups were treated with TRP53 inhibitor (Pifithrin-α hydrobromide, 0.5 pmol/mL (final); 63208-82-2, R&D Systems, Inc, MN, USA), or its activator (NSC 146109 hydrochloride, 0.5 pmol/mL (final), 59474-01-0, R&D Systems, Inc, MN, USA) at 37 ℃. A fifth group was left untreated. Samples were collected at zero, four, and eight hours post treatment (hpt). The *TRP53* level in the samples was determined by dual channel flow cytometry using Phospho-p53 (Cell Signaling Technology, Inc. 8514S) and CD14 antibodies (Cell Signaling Technology, Inc. 29943S) according to the manufacturer’s instructions. The data have been deposited in the Flow Repository database [22] under accession number FR-FCM-Z2JQ.

In order to measure neutrophil gene expression following pre-treatment with RPL39 siRNA, RPL9 siRNA, TRP53 activator, or TRP53 inhibitor and subsequent infection with *Salmonella*, a real-time PCR assay was performed using the SYBR® Green Real-Time PCR Master Mixes (Applied Biosystems™, Inc. 4367659) according to the manufacturer’s instructions. GAPDH and ACTB were used as the house keeping genes. The primer sequences are provided in Additional file 1.

### Intracellular proliferation of *Salmonella* in neutrophils

Primary porcine neutrophils were isolated and infected with *Salmonella* as described above. The intracellular proliferation assay was performed in accordance with a previously established protocol [10, 12]. The neutrophils pre-treated with RPL39 siRNA, RPL9 siRNA, TRP53 activator, TRP53 inhibitor, or left untreated were washed twice and resuspended in PBS. Consequently, 10^6^ viable GFP-labeled *Salmonella* bacteria cells (ATCC® 14028™ GFP) were mixed with neutrophils at a ratio of 100:1, and the mixture of neutrophils and bacteria was incubated at 37 °C, 5% CO_2_ for 2 h. The cells were then rinsed three times with PBS and RPMI 1640 medium (Roswell Park Memorial Institute 1640 Medium), mixed with 100 μg/mL (final) of gentamicin, and incubated for 2 h at 37 °C. The neutrophil cells were collected and then further cultured in 10 μg/mL of gentamicin medium in three replicates for 12 h. Cell samples were collected at 4 h and 8 h following the addition of *Salmonella* to the neutrophils. The bacteria-containing cells (GFP positive) were measured via flow cytometry using the FITC channel according to the manufacturer’s instructions. Data were deposited in the Flow Repository database [22] under accession number FR-FCM-Z2JR.

### Results

### *Salmonella* Typhimurium regulates porcine neutrophil transcriptomes

A total of twelve samples (with the best RNA quality) of infected neutrophils were sequenced, yielding approximately 25 million reads per sample. A total of 14,150 transcripts were covered, with an average sequencing depth of 406X (Additional file 1). The sequencing data was found to contain ~ 1% of *Salmonella* sequences. The result revealed 1,781 transcripts that were differentially expressed between zero and eight hpi (FDR < 0.05 and fold change > 1.5, Table 1 lists the most significant (fdr) transcripts, the full list is available in Additional file 1). The transcripts that were most significantly differentially expressed were encoded by SLC10A1 (down-regulated 83.41-fold), MME (down-regulated 56.41-fold), DOCK4 (down-regulated 38.56-fold), NDUFB2 (up-regulated 30.26-fold), ABI3 (down-regulated 22.13-fold), and CHI3L1 (down-regulated 20.98-fold,). Interestingly, annotation indicated that the expression of a large number of innate immunity-related genes, including IFIT2, IFIT1, ZBP1, MX1, MX2, IFIT3, EIF2KAK2, and IRF7, was down-regulated after eight hours of *Salmonella* infection. Furthermore, 22 ribosomal proteins, including RPL9, RPS14, RPS11, RPL39, MRPS12, MRPS18C, RPL37, and MRPL20, were down-regulated after eight hours of *Salmonella* infection. A total of 68 mitochondrion-related proteins, including GFM1, NT5DC3, MUTYH, SOD1, CYP1B1, CRAT, PRDX4, TIMM44, ACADM, and YWHAG, were up-regulated after eight hours of *Salmonella* infection compared with without infection.

**Table 1:** **Most highly differentially expressed transcripts (top 50) comparing zero and eight hours post** ***Salmonella*** **infection in primary porcine neutrophils**

Gene symbol	Refseq ID	Source	Average expression level (log2)	Fold change	q-value
SLC10A1	XM_001927695	Pig	10.8304	− 83.4142	2.69E−05
MME	XM_003132501	Pig	12.6772	− 56.4129	2.69E−05
DOCK4	XM_021079281	Pig	11.1479	− 38.564	2.69E−05
NDUFB2	NM_001244885	Pig	13.3140	30.25678	2.69E−05
ABI3	XM_003358086	Pig	10.6394	− 22.1323	2.69E−05
CHI3L1	NM_001001540	Pig	12.4107	− 20.9822	2.69E−05
avrA	–	*Salmonella*	12.7307	16.48907	4.81E−05
R3HCC1	XM_021073022	Pig	10.9036	− 11.121	4.81E−05
H2AFY2	XM_021073865	Pig	12.9446	10.55971	4.81E−05
SIPA1L1	XM_005656372	Pig	11.3501	− 7.87188	4.81E−05
sseF	–	*Salmonella*	14.2728	14.12313	4.82E−05
flgM	–	*Salmonella*	6.4344	− 7.50741	4.82E−05
CRYBB3	XM_001929473	Pig	10.4932	− 23.3897	5.71E−05
PLOD2	XM_021069587	Pig	13.0709	128.5496	6.07E−05
DOCK10	XM_003133687	Pig	10.1372	− 23.3319	6.07E−05
CCDC146	XM_021102558	Pig	11.1119	− 10.1757	6.07E−05
SCAPER	XM_021099012	Pig	13.1531	9.680022	6.07E−05
AGO3	NM_001194974	Pig	12.5285	7.508506	6.07E−05
HCAR3	XM_021072989	Pig	13.6520	− 19.7964	6.27E−05
DAB2	XM_021076649	Pig	14.9437	55.62877	6.35E−05
RPS11	NM_001244070	Pig	8.0332	− 8.02668	6.35E−05
rplE	–	*Salmonella*	9.0468	16.20982	6.46E−05
FAAH	NM_213914	Pig	10.5260	− 14.8085	8.45E−05
TP53	NM_213824	Pig	13.3047	8.240427	8.59E−05
TMEM62	XM_021097051	Pig	10.7321	− 12.6827	8.86E−05
HIF1AN	XM_003125588	Pig	10.9592	− 12.4298	9.13E−05
QPCT	XM_003481245	Pig	12.9645	− 12.6145	9.82E−05
MIIP	NM_001244704	Pig	12.8614	9.137871	0.000101
sseD	–	*Salmonella*	13.2127	22.39497	0.000112
PRIM1	NM_001243669	Pig	12.3116	14.28129	0.000112
ERLEC1	XM_003125147	Pig	12.7536	11.4634	0.000112
CCDC40	XM_021066501	Pig	10.7968	− 11.3155	0.000112
SLC39A8	XM_013979001	Pig	11.7795	8.270433	0.000112
pyrH	–	*Salmonella*	8.7281	21.67902	0.000116
RPL9	NM_001243481	Pig	6.2471	− 10.9441	0.000116
ACADM	NM_214039	Pig	11.2439	6.03635	0.000116
FBXL5	XM_013978532	Pig	13.1740	− 35.0706	0.000127
OSGIN2	XM_001925927	Pig	12.3345	− 7.16605	0.000127
KIAA1551	NM_001243821	Pig	12.9331	− 20.2392	0.000132
PLEKHO1	XM_005655399	Pig	11.8700	− 8.81157	0.000143
RPL39	XM_005673863	Pig	6.6511	− 8.66867	0.000143
sopB	–	*Salmonella*	15.0980	18.36901	0.000143
CD69	NM_214091	Pig	10.0376	− 9.70064	0.000143
SERBP1	XM_003127934	Pig	11.5299	8.12229	0.000143
TMEM164	XM_021080001	Pig	11.4298	− 7.43192	0.000143
TMEM123	XM_005667282	Pig	10.0643	− 77.2179	0.000146
MACF1	XM_021095983	Pig	13.7886	12.70162	0.00015
RPL6	NM_001044542	Pig	6.3390	− 7.44624	0.00015
MED7	NM_001044615	Pig	13.3926	11.76379	0.000157
LSM8	XM_003134753	Pig	11.1819	5.565953	0.000159

## The ribosomal pathway was inhibited and the TRP53 pathway was activated in neutrophils following *Salmonella* Typhimurium infection

Pathway analysis indicated that 20 pathways were up-regulated and 25 pathways were down-regulated following *Salmonella* Typhimurium infection (P < 0.005, Table 2). The most down-regulated pathway was the ribosomal pathway, which included 12 down-regulated ribosomal protein genes (Additional file 1). The second most down-regulated pathway was the cytokine-cytokine receptor interaction pathway, which contains 14 differentially expressed genes. The third most down-regulated pathway was the interferon (IFN) α and β signaling pathways, which had 14 differentially expressed genes. There were also 6 mRNA translation-related pathways that were down-regulated, among which the eukaryotic translation initiation pathway, the peptide chain elongation pathway, and the eukaryotic translation termination pathway were most significantly down-regulated. Overall, these data indicated that ribosomal pathways were considerably impacted by *Salmonella* infection.

**Table 2:** **Most highly differentially expressed pathways comparing zero and eight hours post** ***Salmonella*** **infection in primary porcine neutrophils**

Pathway name	Change direction	Pathway genes count	Pathway p-value
MicroRNA (miRNA) biogenesis	Up	5	1.54E−04
Propanoate metabolism	Up	6	4.63E−04
Pyruvate metabolism	Up	7	6.62E−04
Double stranded RNA induced gene expression	Up	4	0.001261
Huntington's disease	Up	15	0.001402
Direct p53 effectors	Up	12	0.001602
Nucleotide Excision Repair	Up	8	0.001777
Pyruvate metabolism	Up	6	0.002333
Regulatory RNA pathways	Up	7	0.002367
mRNA Splicing	Up	12	0.002379
mRNA Splicing—Major Pathway	Up	12	0.002379
Purine catabolism	Up	3	0.002959
Cleavage of Growing Transcript in the Termination Region	Up	6	0.003025
RNA Polymerase II Transcription Termination	Up	6	0.003025
Processing of Capped Intron-Containing PrE−mRNA	Up	12	0.003051
Atm signaling pathway	Up	4	0.003385
RNA Polymerase II Transcription	Up	11	0.003468
HIV Infection	Up	18	0.003569
Pyrimidine metabolism	Up	9	0.004232
Inhibition of TSC complex formation by PKB	Up	2	0.004779
Ribosome	Down	12	3.01E−05
Cytokine-cytokine receptor interaction	Down	14	9.58E−05
Interferon alpha/beta signaling	Down	6	1.10E−04
Eukaryotic Translation Termination	Down	9	3.07E−04
Peptide chain elongation	Down	9	3.07E−04
Viral mRNA Translation	Down	9	3.55E−04
Nonsense Mediated Decay (NMD) independent of the Exon Junction Complex (EJC)	Down	9	4.67E−04
Eukaryotic Translation Elongation	Down	9	4.67E−04
L13a-mediated translational silencing of Ceruloplasmin expression	Down	10	5.85E−04
GTP hydrolysis and joining of the 60S ribosomal subunit	Down	10	6.55E−04
Formation of a pool of free 40S subunits	Down	9	9.93E−04
Cap-dependent Translation Initiation	Down	10	0.001121
Eukaryotic Translation Initiation	Down	10	0.001121
Class A/1 (Rhodopsin-like receptors)	Down	16	0.00113
Nonsense Mediated Decay (NMD) enhanced by the Exon Junction Complex (EJC)	Down	9	0.001556
Nonsense-Mediated Decay (NMD)	Down	9	0.001556
Acyl chain remodelling of PI	Down	2	0.001704
Acyl chain remodelling of PS	Down	2	0.001704
Binding and entry of HIV virion	Down	2	0.001704
SRP-dependent cotranslational protein targeting to membrane	Down	9	0.002351
Influenza Viral RNA Transcription and Replication	Down	9	0.002855
Signaling by GPCR	Down	24	0.004493
Influenza Life Cycle	Down	9	0.004493
Influenza Infection	Down	9	0.004895
Oxygen-dependent asparagine hydroxylation of Hypoxia-inducible Factor Alpha	Down	2	0.004973

There were also several up-regulated pathways in the neutrophils following *Salmonella* infection (Table 2). The top three were microRNA (miRNA) biogenesis, propanoate metabolism, and pyruvate metabolism. Interestingly, the TRP53 pathway, which included 12 differentially expressed genes, was also significantly up-regulated. Among the differentially expressed ribosomal protein genes, RPS11, RPL9, and RPL39 were the most significant (Table 1 and Additional file 1). Interestingly, RPL39 and RPL9 are known to be highly associated with *TRP53* levels [20]. RPL39 and RPL9 encode the ribosomal protein L39 and L9, whereas TRP53 encodes the tumor protein p53, which is an important regulator in the intracellular proliferation of *Salmonella enterica* serovar Typhimurium LT2 in pigs [10]. Therefore, RPL39, RPL9, and TRP53 were selected for further validation in the following analysis.

### Silencing of RPL39 and RPL9 caused up-regulation of *TRP53* level in neutrophils

**Figure 1:**
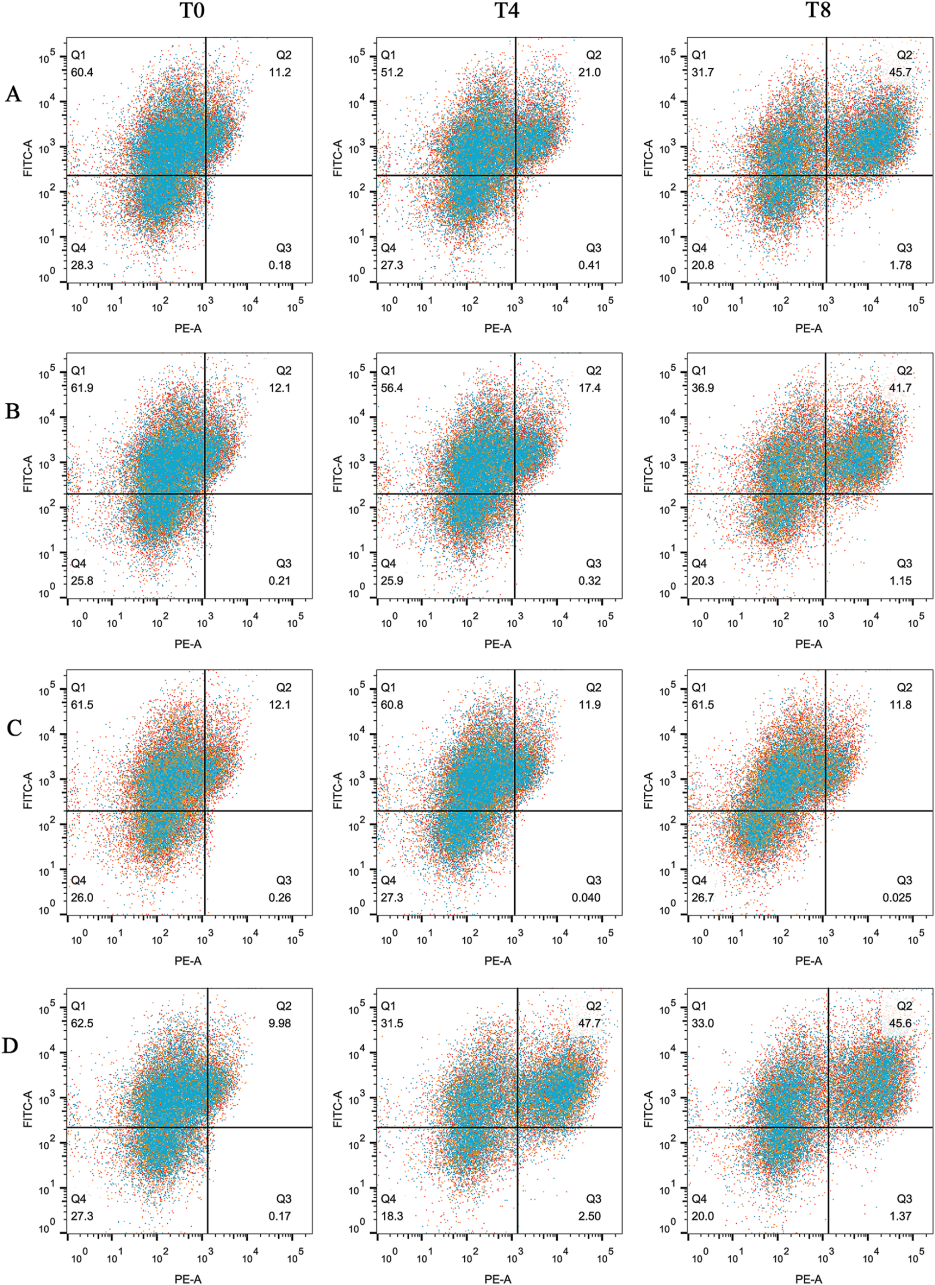
**Reducing RPL39 and RPL9 expression through siRNA knock-down up-regulates *TRP53*** **expression in neutrophils.** Flow cytometry plots for rested and stimulated neutrophils following antibody staining for *TRP53* (FITC-A) and *CD14* (PE-A). Neutrophils were either untreated at zero hours (column T0), treated for four hours (column T4), or treated for eight hours (column T8) with RPL39 siRNA (row **A**), RPL9 siRNA (row **B**), TRP53 inhibitor (row **C**), or TRP53 activator (row **D**).

Primary pig neutrophils were left untreated (Figure 1T0), or were treated with RPL39 siRNA, RPL9 siRNA, or were stimulated with TRP53 inhibitor or activator for four hpt (Figure 1T4) and eight hpt (Figure 1T8). *TRP53* levels in each treatment were monitored via flow cytometry. Three subsets of cell populations emerged from the analysis of CD14/TRP53 in neutrophils at eight hpt: CD14^high^/TLR2^high^, CD14^low^/TRP53^low^, and CD14^high^/TLR2^low^ (Figure 1T8). The percentage of CD14^high^/TLR2^high^ cells was increased in RPL39 knock-down neutrophils at both four and eight hpt (P < 0.05). However, the proportion of CD14^low^/TRP53^low^ cells remained almost unchanged (Figure 1A). The percentage of CD14^high^/TLR2^high^ cells in *RPL9* knock-down neutrophils remained unchanged at four hpt but was increased at eight hpt (P < 0.05). The proportion of CD14^low^/TRP53^low^ cells after the RPL9 siRNA treatment remained almost unchanged at four hpt but was decreased at eight hpt (P < 0.05, Figure 1B). All three subset populations remained almost unchanged at both four and eight hpt after the TRP53 inhibitor treatment (Figure 1C). The percentage of CD14^high^/TRP53^high^ cells was increased by 50% in samples exposed to the TRP53 activator at both four hpt and eight hpt, compared with the zero hour controls (Figure 1D). At four hpt, the CD14^high^/TLR2^high^ population treated with the TRP53 activator was twice as large as the RPL39 and RPL9 silenced neutrophils. The neutrophils treated with the TRP53 inhibitor had the lowest CD14^high^/TLR2^high^ population at four hpt. At eight hpt, the CD14^high^/TLR2^high^ cell population was increased when treated with the TRP53 activator, RPL39 siRNA, and RPL9 siRNA. The CD14^high^/TLR2^high^ cell population remained the lowest when treated with the TRP53 inhibitor. Therefore, treatment with RPL39 siRNA and RPL9 siRNA enhanced TRP53 expression, confirming their inverse association.

### RPL39 and RPL9 siRNA treatment activated genes associated with cell cycle arrest and apoptosis and inhibited innate immunity genes

**Figure 2:**
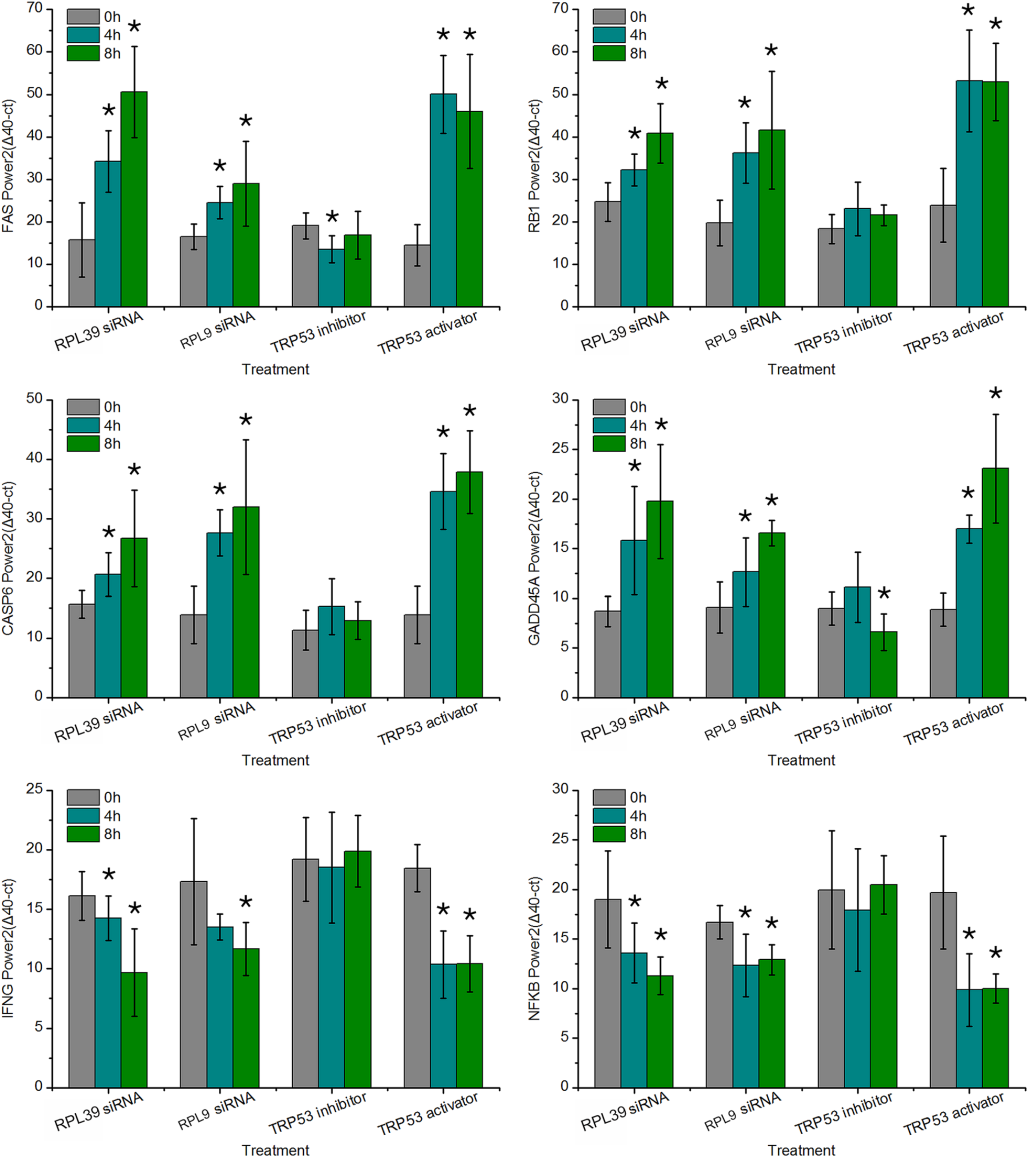
**Modulation of the ribosomal/TRP53 pathway increases evidence of apoptosis and immune system arrest.** The transcription level of four apoptosis genes and two innate immunity genes were measured via RT-PCR following pre-treatment with TRL39 siRNA, RPL9 siRNA, TRP53 inhibitor, or TRP53 activator and infection with *Salmonella*. Gene expression was normalized to GAPDH transcription level ${2}^{\Delta 40-\mathrm{ct}}$ for **A** FAS, **B** RB1, **C** CASP6, **D** GADD45A, **E** NFKB, and **F** INFG. Error bars represent mean ± SD. * = P < 0.05 (ANOVA test, number of replicates = 3).

The transcription level of four TRP53 target genes (FAS, RB1, CASP6, and GADD45A) and two innate immunity genes (NFKB and IFNG) were investigated in neutrophils following the treatments and infections described above. The transcription level of FAS increased after exposure to the TRP53 activator, RPL39 siRNA, and RPL9 siRNA at both four and eight hpt (P < 0.05, Figure 2A). The transcription level of FAS in neutrophils treated with the TRP53 inhibitor remained unchanged at eight hpt. The transcription level of RB1 in neutrophils treated with RPL39 and RPL9 siRNA at four and eight hpt was double the zero hour control sample (Figure 2B), whereas the transcription level of RB1 in neutrophils treated with the TRP53 activator at four and eight hpt was approximately 2.5-fold higher than in the zero hour control. Our results also showed that the transcription level of RB1 in neutrophils treated with the TRP53 inhibitor remained almost the same as in the control samples. Similar to FAS and RB1, the transcription profile of CASP6 increased in neutrophils treated with RPL39 siRNA after four hours and was increased in RPL9 siRNA and TRP53 activator-treated neutrophils at both four and eight hpt (P < 0.05, Figure 2C). The transcription level of CASP6 in neutrophils treated with the TRP53 inhibitor remained stable at both four and eight hpt. We also found that the transcription levels of GADD45A in neutrophils treated with RPL39 siRNA, RPL9 siRNA, and TRP53 activator were significantly higher than in non-treated controls at both four and eight hpt (P < 0.05, Figure 2D). Conversely, the transcription level of GADD45A in the TRP53 inhibitor-treated samples at eight hpt was lower than in the controls. The transcription levels of IFNG in RPL39 siRNA, RPL9 siRNA, and TRP53 activator-treated neutrophils were significantly lower than in the non-treated controls at both four and eight hpt (P < 0.05, Figure 2E). The expression profile of NFKB was shown to be similar to IFNG, and expression was inhibited in most of the RPL39 siRNA, RPL9 siRNA, and TRP53 activator-treated samples (Figure 2F). However, at four and eight hours post TRP53 inhibitor treatment, the level of NFKB and IFNG mRNA remained almost the same as in non-treated controls.

### The ribosomal/TRP53 pathway is likely to be associated with intracellular proliferation of *Salmonella* in infected neutrophils

**Figure 3:**
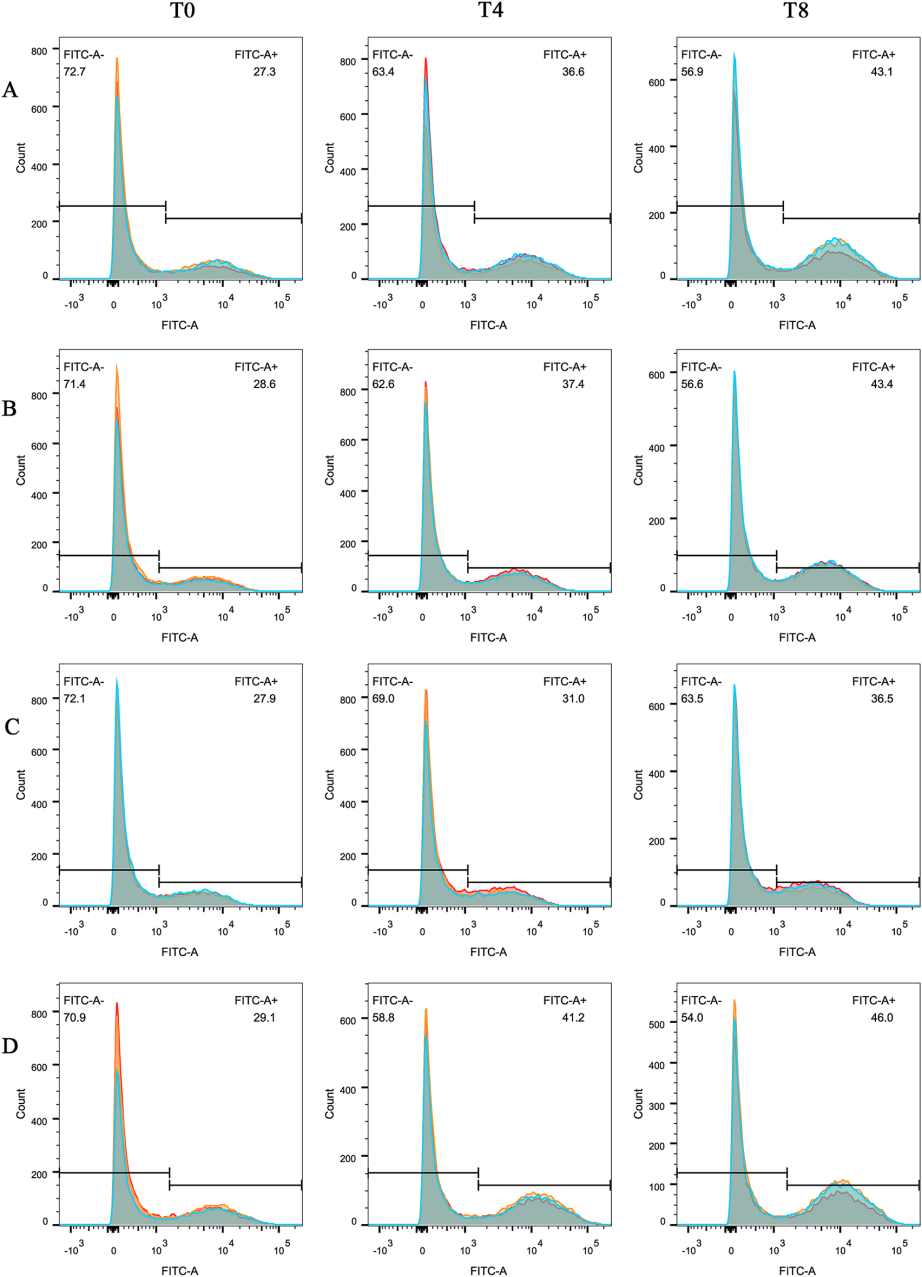
**Modulation of neutrophil ribosomal/TRP53 pathway impacts the ability of** ***Salmonella*** **to infect and proliferate intracellularly.** Flow cytometry for neutrophils left untreated at zero hours (column T0) or pre-treated for four (column T4) or eight hours (column T8) with RPL39 siRNA (row **A**), RPL9 siRNA (row **B**), TRP53 inhibitor (row **C**), and TRP53 activator (row **D**) and then infected with GFP fluorescent labeled *Salmonella*. Fluorescence intensity represents bacterial replication. Line graphs show the overlay of three replicate experiments.

Porcine neutrophils were left untreated (Figure 3T0) or were pre-treated with RPL39 siRNA, RPL9 siRNA, a TRP53 inhibitor, or a TRP53 activator before infection with GFP-labeled *Salmonella* for four (Figure 3T4) and eight hours hpt (Figure 3T8), during which intracellular *Salmonella* counts were monitored by flow cytometry. The percentage of GFP^pos^ (GFP-positive) cells was increased when treated with RPL39 siRNA at both four and eight hpt (P < 0.05), and the percentage of GFP^pos^ cells was approximately 15% higher at eight hpt than at four hpt (Figure 3A). The percentage of GFP^pos^ cells was also increased in RPL9 siRNA-treated neutrophils at both four and eight hours (P < 0.05), with the percentage of GFP^pos^ cells being 13% higher at eight compared with at four hpt (Figure 3B). Treatment with the TRP53 inhibitor presented with the lowest GFP^pos^ cells (approximately 30%) at both four and eight hpt, indicating a lower number of bacteria (Figure 3C). At eight hpt, fluorescence had increased the most (45%) in GFP^pos^ cells treated with the TRP53 activator, indicating a high level of intracellular *Salmonella* (Figure 3D)*.*

### Discussion

Previous studies have demonstrated that *Salmonella enterica* serovar Typhimurium infection in piglets can lead to the inhibition of the transcription of ribosomal genes in peripheral blood [9, 10]. This has been confirmed in our study, wherein infection with *Salmonella* was shown to inhibit the transcription of 12 ribosomal genes in primary porcine neutrophils. This decrease in ribosomal transcription will strongly impact the activity of the ribosomal protein machine, and the initiation, extension, and/or termination of translation will be blocked, and finally will lead to the reduction in protein synthesis. Here, we found that the ribosomal protein genes RPL39 and RPL9 were highly down-regulated when neutrophils were infected with *Salmonella*. RPL39 and RPL9 are central protuberance-specific assembly factors that are the most important ribosomal proteins contributing to the maintenance of normal nucleolar structure. Therefore, the knock-down of RPL39 and RPL9 can prevent the formation of a normal nucleolar structure [20]. Many ribosomal proteins are likely involved in the regulation of *TRP53.* Our data indicate that the decrease in RPL39 and RPL9 results in the up-regulation and accumulation of *TRP53* protein in neutrophils.

Our results indicate that the *Salmonella-*mediated inhibition of RPL39 and RPL9 can increase the expression of FAS, RB1, CASP6, and GADD45A in a TRP53-dependent manner. We also found that two innate immunity genes, INFG and NFKB, were significantly inhibited by RPL39 siRNA, RPL9 siRNA, and TRP53 activator treatment. FAS encodes a protein containing a death domain and plays a central role in the physiological regulation of programmed cell death. FAS has also been implicated in the pathogenesis of various malignancies and immune diseases [23]. The interaction of this receptor with its ligand leads to the formation of a death-inducing signaling complex that triggers a downstream caspase cascade, leading to apoptosis [24]. RB1 encodes a protein that is a negative regulator of the cell cycle process and acts by binding and regulating the transcription factor E2F1s [25, 26]. CASP6 encodes a caspase protein, which plays a central role in the execution-phase of cell apoptosis [27]. GADD45A is an important factor that maintains the cells in stressful and growth arrest conditions [28]. FAS, RB1, CASP6, and GADD45A are downstream of the TRP53 cascade, and activation of these genes would lead to cell cycle arrest and apoptosis. Therefore, we hypothesize that inhibition of ribosomal genes and activation of apoptosis could be an important mechanism for *Salmonella* to regulate intercellular conditions, promoting survival and replication within neutrophils. Furthermore, the inhibition of NFKB and IFNG transcription and the down-regulation of the TNF pathway indicate that the infected neutrophils have decreased immune function, which would provide a niche for *Salmonella* survival and replication.

TRP53 is a tumor suppressor gene that balances cell survival and death in response to a variety of intrinsic and extrinsic stress signals [29, 30]. Infection with *Salmonella enterica* serovar Typhimurium LT2 has been demonstrated to increase the *TRP53* level in the serum of piglets [10], which could induce cell-cycle arrest or apoptosis [31, 32]. It has been shown that TRP53 activity can be induced in a type I IFN-dependent manner [33]. However, our results indicate that the IFN-α/INF-β signaling was significantly inhibited in *Salmonella*-infected neutrophils, which suggests that TRP53 was activated by effectors other than IFNs. TRP53 can direct E2F2-mediated growth arrest, which involves the target gene GADD45A [34]. GADD45A has previously been shown to be up-regulated in *Salmonella*-challenged pigs [10] and is currently shown to be up-regulated in *Salmonella*-infected neutrophils. This further supports the hypothesis that during *Salmonella* infection, the cell cycle of neutrophils is arrested and apoptosis is promoted.

Here, we showed that the knock-down of *RPL39 and RPL9*, or the activation of TRP53, could promote intracellular *Salmonella* proliferation in neutrophils. We speculate that this impact on *Salmonella* growth is caused by favoring a cell cycle arrested state, allowing the bacteria to proliferate more successfully. This is similar to other documented cases where bacterial pathogens block cell cycle progression, which leads to favorable conditions for colonization and dissemination [35, 36].

### Conclusion

**Figure 4:**
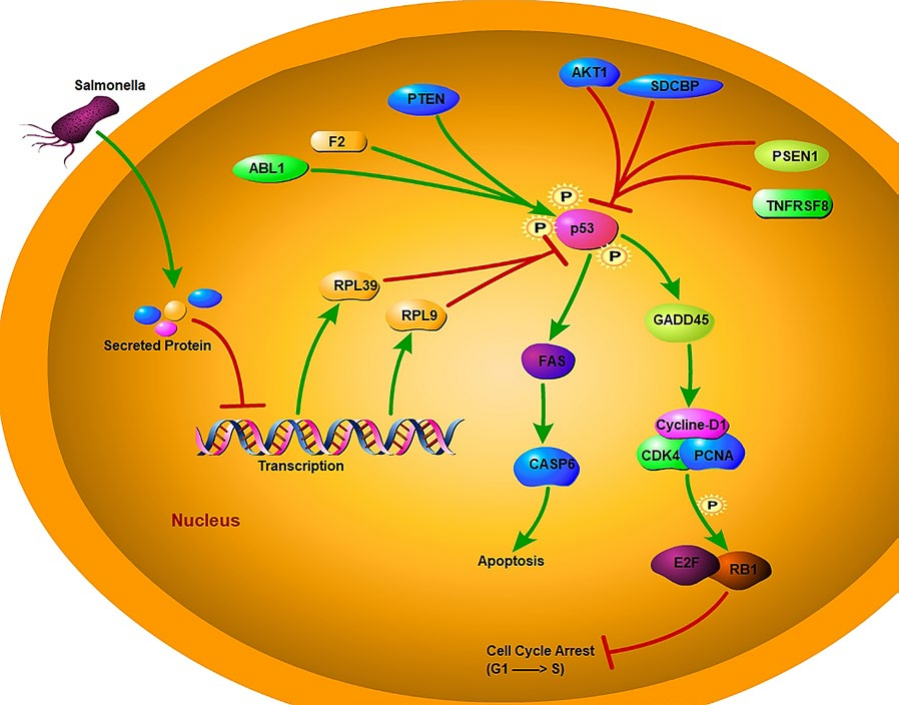
**Ribosomal/TRP53 pathway pattern mediated by Salmonella infection.** Following the intracellular invasion of *Salmonella*, the bacterium secretes proteins that inhibit the transcription of RPL39 and RPL9, and then increases the accumulation of *TRP53*. TRP53 acts on the FAS/CASP6 sub-pathway and promotes apoptosis. TRP53 can also modulate the GADD45A sub-network and causes cell cycle arrest. Besides RPL39 and RPL9, three genes (ABL1, F2, and PTEN) which promote TRP53 activity, and four genes (AKT1, SDCBP, PSEN1, and TNFRSF8) which inhibit TRP53 activity were differentially expressed in neutrophils infected with *Salmonella*. The regulatoryrelationships shown in this figure were either discovered in this study or curated from gene expression regulation relationships deposited in GEREDB database [18].

This study described the inhibition of RPL39 and RPL9 transcription following *Salmonella* infection, which leads to the activation of *TRP53*. This ribosomal/TRP53 pathway is an important factor that influences the intracellular proliferation of *Salmonella* counts in neutrophils (summarized in Figure 4). However, detailed mechanisms of how *Salmonella* regulates the transcription level of ribosomal genes underlying this observation remain to be discovered.

## Supplementary information

**Additional file 1.** Sequencing matrix, the information on total number of reads, sequencing depth, coverage, processed data, and etc.

## Data Availability

The RNAseq data discussed in this study were deposited in the NCBI GEO database [19] under the accession number GSE148236. The flow cytometry data were deposited in the Flow Repository database [22] under accession number FR-FCM-Z2JQ and FR-FCM-Z2JR.

## Abbreviations

cfu: colony forming units
FDR: false discovery rate
hpi: hours post infection
hpt: hours post treatment
RIN: rNA Integrity Number
ROS: reactive oxygen species
